# Circulating Potassium/Magnesium Ratio, Thyroid Stimulating Hormone, Fasting Plasma Glucose, Oxidized LDL/Albumin Ratio, and Urinary Iodine Concentration Are Possible Entities for Screening for Preeclampsia in Low-Resource Settings

**DOI:** 10.3390/medicina61040600

**Published:** 2025-03-26

**Authors:** Charles Bitamazire Businge, Benjamin Longo-Mbenza, Andre Pascal Kengne

**Affiliations:** 1 Department of Obstetrics and Gynaecology, Faculty of Medicine and Health Sciences, Walter Sisulu University, Mthatha 5117, South Africa; 2 Faculty of Medicine, University of Kinshasa, Kinshasa, Democratic Republic of the Congo; longombenza@gmail.com; 3 Department of Public Health, Lomo University of Research, 652 Freesias, Kinshasa, Democratic Republic of the Congo; 4 African Population Health Research Center (APHRC), Nairobi 00100, Kenya; akengne@aphrc.org

**Keywords:** preeclampsia, biomarkers, potassium/magnesium ratio, urinary iodine excretion, FPG, TSH

## Abstract

*Background and Objectives*: Several micro- and macro-nutrient malnutrition states that are routinely assessed during clinical care of women in the antenatal period have been proposed as risk factors for preeclampsia. However, there is a paucity of data on the potential use of these biomarkers for detection of preeclampsia. The aim of this case-control study was to investigate the association of biomarkers from routine clinical tests, and those specific to micro- and macro-nutrient malnutrition, with the risk of preeclampsia. *Materials and Methods*: Venous blood samples of 250 participants with preeclampsia and 150 pregnant women without preeclampsia were collected and assayed immediately for the full blood count, urea and electrolytes, high-density cholesterol (HDL), total cholesterol, triglycerides, low-density lipoprotein cholesterol (LDL), oxidized low-density lipoprotein cholesterol (OxLDL), and selenium, in addition to urine iodine concentration (UIC). *Results*: The serum potassium/magnesium ratio (K^+^/Mg^2+^), UIC, fasting plasma glucose (FPG), thyroid stimulating hormone (TSH), lymphocyte percentage (L/WBC%), and the oxidized LDL/albumin ratio (OxLDL/Alb) were identified as independent predictors of preeclampsia. *Conclusions*: Serum potassium/magnesium ratio and other analytes essential for various biological processes, some of which are assayed during routine care, were significantly associated with preeclampsia, warranting further exploration as potential screening biomarkers in low-resource settings.

## 1. Introduction

Preeclampsia complicates 3 to 8% of all pregnancies and is one of the major causes of perinatal mortality and morbidity [1]. In the long term, it also accounts for an increased risk of premature cardiovascular diseases in both mothers and their offspring, secondary to the structural modification of proteins, lipids, and epigenetic changes that affect DNA expression. Several risk factors for preeclampsia have been identified. These include a maternal or family history of preeclampsia or hypertension, ethnicity, extreme maternal age, primiparity, primipaternity, in vitro fecundation (IVF), multiple pregnancy, obesity, and systemic disorders such as diabetes mellitus (DM), kidney disease, and autoimmune diseases [1]. In an attempt to identify women at high risk of preeclampsia, several biomarkers have been studied. These include serum and plasma markers of placental function, endothelial dysfunction, renal dysfunction, general metabolic status, oxidative stress, and haemolysis and inflammation. However, most biomarkers have yielded low sensitivity and specificity for the prediction of preeclampsia [2,3,4]. So far, the main predictive markers are those that are associated with placental function, which include the placental growth factor (PlGF) and soluble Fms-like tyrosine kinase 1 (sFlt-1) [2,3,4,5]. PlGF has been found to identify women at risk of preeclampsia from as early as 11 weeks of amenorrhoea (WOA), while sFlt-1 is more predictive of preeclampsia among women with a gestation age ≥ 20 WOA [6,7,8,9,10,11,12,13].

Previous studies have reported that micronutrients (selenium and iodine deficiency) and macronutrient malnutrition (obesity) are risk factors for preeclampsia [14,15,16]. The primary objective of the current study was to investigate the biomarkers from routine blood tests, and those specific to micro- and macro-nutrient nutrition, associated with the risk of preeclampsia. The secondary objective was to ascertain cut-off levels of these biomarkers that can potentially be used for the identification of pregnant women at risk of incident preeclampsia.

## 2. Materials and Methods

### 2.1. Study Design

The current study design is an unmatched case-control study design of 150 normotensive pregnant women at term and 250 women with incident preeclampsia. This study was carried out as a secondary analysis of archived data from a matched case-control study of pregnant normotensive controls at term and cases of preeclampsia. They were part of the Communicable Disease, Nutritional, Environmental Epidemiology, and Cardio-metabolic Risk Study conducted in Kinshasa Province, the Democratic Republic of the Congo, between 2007 and 2008 at Lomo Medical Centre, Kinshasa Limete.

### 2.2. Study Population

The target population for the primary study comprised women who attended maternity care at Lomo Medical Centre in Limete, a peri-urban suburb of Kinshasa, the Democratic Republic of the Congo. One hundred and fifty women who remained normotensively pregnant till delivery at term were enrolled in the primary study as controls. This method of control selection was chosen to avoid the probable misclassification of women who could have developed late-onset preeclampsia in the later part of the third trimester as controls. Three hundred cases of preeclampsia without other co-morbidities were included. In the primary study, 50 of the 300 cases of preeclampsia enrolled in the primary study were referred to a tertiary hospital before the completion of data collection and hence had incomplete data.

### 2.3. Inclusion and Exclusion Criteria for the Current Study

The inclusion criteria for this secondary analysis were participants of the primary study with no missing data. Participants who remained normotensive till delivery at term were classified as controls. The cases were preeclamptic women with no missing data enrolled in the primary study soon after diagnosis of preeclampsia. All primary study participants who had missing data were excluded, thus making the current study an unmatched case-control study of 150 normotensive controls and 250 cases of preeclampsia.

The rationale for having controls at term was that women who are normotensive till delivery are likely to have maintained physiological levels of the different nutritional biomarkers under investigation. Those who develop preeclampsia may have had higher or lower levels of these micro/macronutrients before the index pregnancy, or these could have become excessive or deficient during pregnancy. They may remain without any features of preeclampsia (i.e., subclinical) for some of the gestational period. Some may manifest with preeclampsia at any gestational age after serum levels or urinary levels of specific biomarkers exceed or diminish beyond a given cut-off value. In some circumstances, the regular physiological interaction between two or more nutrients may influence or modify the cut-off value.

### 2.4. Methods

Pre-eclampsia was defined according to the International Society for the Study of Hypertension in Pregnancy [17]. Participants were diagnosed with pre-eclampsia when they presented with new onset of hypertension (>140 mmHg systolic (SBP) and or >90 mmHg diastolic (DBP) blood pressure) after 20 weeks gestation with proteinuria (spot urine protein/creatinine >30 mg/mmol, or >300 mg/day or 2+ on dipstick testing) or other maternal organ dysfunction: renal insufficiency (creatinine >90 μmol/L; 1.02 mg/dL); liver involvement (elevated transaminases (at least twice upper limit of normal ± right upper quadrant or epigastric abdominal pain)), neurological complications (altered mental status, blindness, stroke, hyperreflexia, severe headaches, and/or persistent visual scotomata), haematological complications (thrombocytopenia (platelet count below 150,000/dL, disseminated intravascular coagulation or haemolysis)), and uteroplacental dysfunction (foetal growth restriction, abruptio placentae, or intrauterine foetal death). Participants were diagnosed with severe pre-eclampsia when they presented with SBP > 160 mmHg or DBP > 110 mmHg with or without systemic organ involvement. Participants were diagnosed with eclampsia when they presented with SBP > 140 mmHg or DBP > 90 mmHg after 20 weeks gestation, accompanied by convulsions.

Trained nurses measured height, weight, waist circumference (WC), SBP, and DBP to standardized procedures. Blood pressure was measured according to the American Heart Association guidelines, with the patient’s elbow flexed at the heart level. The average of the two measurements with a standard mercury sphygmomanometer taken at intervals ≥ 2 min after the participants had been sitting for at least 30 min was used [18].

Venous blood was collected from the cubital fossa between 7:00 and 9:00 a.m. into ethylenediaminetetraacetic acid (EDTA) and sodium fluoride (NaF) vacutainers. The samples were processed and assayed immediately to measure the full blood count (FBC), urea and electrolytes (U&E), the concentrations of high-density cholesterol (HDL), total cholesterol, triglycerides, low-density lipoprotein cholesterol (LDL), oxidized low-density lipoprotein cholesterol (oxLDL), C-peptide, and glucose. Laboratory data were obtained using calibrated and standard routine procedures and specific protocols of the manufacturers’ such as the CyFlowR Counter (Partec GmH, Munster, Germany), the Hydrasys system (Serbia, Evry, France), a spectrophotometer (Hospital Diagnostics, Florence, Italy), kits from Biome’rieux (Marcy l’Etoile, France), the Mercodia AB (Silveniusgatan 8 A, SE754, Uppsala, Sweden), and a caloric Sensor Hach DR/2010 spectophotometer (HACH, Loveland, CO, USA). TSH was measured by an enzyme-linked immunosorbent assay method purchased from DIALAB GmbH IZ-NOE Sued Company (Wiener Neuforf, Austria), and an Objekt M55 (Wiener Neudorf, Austria). NO was measured using Cayman kits (Cayman Chemical Company, Ann Arbot, MI, USA). Urinary iodine concentration was measured using the Sandell–Kolthoff method.

Variables studied as possible predictors of preeclampsia included morphological markers, such as waist circumference (WC), and metabolic syndrome (MS) markers, such as hip circumference (HC) and systolic and diastolic blood pressure, while laboratory data were circulating markers or biomarkers associated with metabolic syndrome, which included: triglycerides, total cholesterol, HDL, LDL, glucose, and uric acid. Additional biomarkers included: NO for endothelial dysfunction; vitamin C for exogenous anti-oxidants; lymphocytes percent, serum ferritin, anti-Helicobacter pylori IgG, GGT, and CRP for inflammation, infections, and cytokines; selenium deficiency a key trace element in nutrition, OxLDL for oxidative stress imbalance and atherosclerosis; cortisol as a distress hormone; and UIC and TSH for iodine nutrition and thyroid hormones.

### 2.5. Statistical Analysis

Proportions of categorical variables were compared using the chi-square test, while means of continuous variables were compared using Student’s *t*-test or ANOVA. The Kruskal–Wallis test was used to compare the medians of non-normally distributed data. A *p*-value of <0.05 was considered statistically significant. Logistic regression was carried out to identify the biomarkers that can independently predict preeclampsia in the study population. The diagnostic performance of these biomarkers at discriminating preeclamptic participants and normotensive controls was tested using the receiver operating characteristic curve (ROC). The areas under the curve (AUCs), with the corresponding 95% confidence intervals, the standard error, and the sensitivity and specificity at the optimal cut-off values of the candidate diagnostic biomarkers were calculated. These optimal cut-offs were derived using the Youden index method. All analyses were performed using the Statistical Package for Social Sciences (SPSS) for Windows version 23.0 (SPSS Inc., Chicago, IL, USA).

## 3. Results

### 3.1. General Characteristics

The median (IQR) age was 33 (29–37.5) years for cases and 33.5 (33.5–37) years for controls (*p* = 0.135). The median (IQR) gestational age (weeks of amenorrhea) at recruitment and sample collection was 32 (24–37.5) for cases and 39 (38–39) for controls (*p* < 0.001).

### 3.2. Biomarkers Associated with Preeclampsia

Table 1 summarizes the median (25th and 75th percentile) serum values of several biomarkers for cases and controls. This univariate analysis showed higher levels (*p* < 0.05) of low-density cholesterol (LDL), oxidized low-density cholesterol (OxLDL), triglycerides (TG), total cholesterol (TC), waist circumference (WC), hip circumference (HC), body mass index (BMI), fasting plasma glucose (FPG), cortisol, TSH, T3, T4, OxLDL/albumin ratio, γ-glutamyl transferase (GGT), C-reactive protein (CRP), and potassium/magnesium ratio among cases of preeclampsia compared to controls.

The levels of vitamin C, selenium, nitric oxide (NO), lymphocytes, serum magnesium, and urinary iodine concentration (UIC) were lower (*p* < 0.05) among cases of preeclampsia than controls. However, the serum HDL and potassium levels were not significantly different (*p* ≥ 0.05) between cases of preeclampsia and normotensive controls.

### 3.3. Independent Predictors of Preeclampsia

All biomarkers that were significantly associated with preeclampsia on univariate analysis were entered into the binary logistic model. After adjustment for confounding factors (all variables not maintained in the equation), only the OxLDL/albumin ratio, lymphocyte percentage, UIC, K^+^/Mg^2+^ ratio, TSH, and FPG were identified as significant predictors of preeclampsia (Table 2).

### 3.4. The AUC, Optimal Thresholds, Sensitivity, and Specificity of the Independent Predictors of Preeclampsia

The AUC, optimal thresholds, sensitivity, and specificity at the optimal threshold of those significant biomarkers, are shown in Table 3. The AUC ranged from 0.75 (95% CI 0.69–0.80) for the OxLDL/Alb ratio to 0.97 (0.95–0.99) for K^+^/Mg^2+^. Sensitivities at the optimal thresholds ranged from 60% for nitric oxide to 98% for UIC, and specificity from 63.2% for % lymphocytes to 96% for selenium.

## 4. Discussion

In the current study, the analytes identified as independent predictors of preeclampsia were the oxidized LDL/albumin ratio, lymphocyte levels, urine iodine concentration, serum potassium/magnesium ratio, TSH, and FPG. This suggests the need for closer attention to the role of a high energy–low protein diet, iodine deficiency, subclinical hypothyroidism, magnesium deficiency, and gestational diabetes in the increased risk of preeclampsia in the study population.

Preeclampsia is a multisystem disorder for which clinical symptoms and signs alone cannot adequately predict adverse maternal and foetal outcomes [19]. Previous research in high-resource settings has revealed the potential utility of placental-derived factors for predicting and early diagnosing preeclampsia, especially the sFlt-1/PlGF ratio [20,21]. Of the variables identified as independent predictors of preeclampsia in the current study, the serum potassium/magnesium ratio had the highest area under the curve. Therefore, the serum potassium/magnesium ratio, which is more affordable compared to the sFlt-1/PlGF ratio, is potentially a candidate screening test for women at high risk of preeclampsia in low-resource settings, if our results can be confirmed by future studies as recommended by the World Health Organization [22].

In the current study, the median, 25th, and 75th percentile levels of serum magnesium were not only significantly reduced among preeclamptic women compared to normotensive controls but were also much lower than the lower limit of the normal serum magnesium level of 0.74–0.95 mmol/L [23,24]. The median serum potassium levels were comparable (Table 1) between cases and controls, implying that the relationship between the pregnant woman’s potassium and magnesium levels may determine the risk of preeclampsia. Low dietary intake is the leading known cause of hypomagnesemia [25,26]. Globally, the prevalence of hypomagnesemia is between 2.5–15%, despite the availability of foods rich in magnesium, including whole grains, leafy vegetables, and nuts [26]. It is estimated that about 85% of magnesium is lost during food processing, putting populations dependent on processed foods at high risk of hypomagnesemia, especially in the era of nutrition transition and urbanization sweeping across the developing world [27].

Several other researchers found lower serum magnesium levels with no significant difference in serum potassium among women with preeclampsia compared to controls [28,29,30,31,32,33]. Our findings and previous research suggest that the potassium/magnesium ratio may be a good biomarker for predicting incident preeclampsia at any gestation age. The high diagnostic potential of the serum potassium/magnesium ratio for preeclampsia, with a specificity and sensitivity in the current study similar to that of the sFlt-1/PlGF ratio [5,20], makes it a potential candidate biomarker warranting further exploration for use in resource-limited resource settings and populations at risk. Moreover, both magnesium and potassium are excreted in the urine, which provides an opportunity for assessing whether the urinary magnesium/potassium ratio may show similar diagnostic performance, which may pave the way for even a more affordable urine dipstick potassium/magnesium screening test for preeclampsia that can be used in primary health care clinics.

Magnesium is required to maintain physiological cellular potassium levels [34]. Magnesium is a cofactor of the Na^+^/K^+^ ATPase, whose malfunction is secondary to hypomagnesemia and results in the depletion of intracellular K+ and accumulation of intracellular Na^+^. This stimulates the Na+–Ca^2+^ pump exchange activity, resulting in high intracellular Ca^2+^ with resultant vasoconstriction, leading to hypertension [35,36,37]. Secondly, in vivo and in vitro studies have confirmed that inadequate magnesium intake/hypomagnesaemia leads to endothelial dysfunction, oxidative stress, insulin resistance, and hyperlipidaemia, which are known mechanisms in the pathology of preeclampsia and precursors of atherosclerosis [27,38]. Magnesium deficiency increases the transport of low-density lipoproteins across the endothelium, whose accumulation in the sub-endothelial space is associated with incident atherosclerosis [38,39]. Hence, hypomagnesaemia coupled with a high-energy diet or obesity may multiply the risk of preeclampsia and cardiovascular disease. Indeed, in one study it was found that carotid intima–media thickness, an early marker of atherosclerosis and cardiovascular disease, was significantly higher among preeclamptic women when compared to normotensive controls [14].

In the current study, the mean urine iodine excretion (UIC) was the biomarker with the second-best diagnostic potential for preeclampsia after the serum K^+^/Mg^2+^ ratio. At a cut-off of 239 µg/L, UIC had a sensitivity of 98% and specificity of 80% for the diagnosis of preeclampsia. Previous studies have also shown an association between UIC and preeclampsia [40,41,42]. Although the standard practice has been the use of median UIC of school-age children (SAC) for the identification of populations at risk of inadequate iodine intake, SAC UIC is not usually representative of pregnant women and other population groups at high risk of iodine deficiency [43,44]. Some have found that there is a daily variation in UIC concentration, making it a less accurate measure of iodine nutrition for individualized assessment [45,46,47]. Rasmussen et al. found that the fasting UIC tends to underestimate the 24 h UIC compared to samples taken later in the day [48]. However, all UIC values taken at various times of the day were highly correlated with the 24 h UIC, with a correlation coefficient (r) ranging between 0.61–0.74.

About 70 µg of iodine is required for daily thyroid hormone synthesis in an iodine-replete individual with sufficient intra-thyroid iodine storage of 15–20 mg [49]. In physiological pregnancy, the net daily iodine requirements increase to about 120 µg. In chronically iodine-deficient individuals with intra-thyroid iodine storage of about 2–5 mg, compensatory mechanisms will increase thyroid iodide trapping by about 50% [49]. In women with moderate to severe iodine deficiency in pregnancy, most of the iodine consumed will be taken up by the thyroid gland, the placenta, and the foetus leading to low serum iodide levels. Hence, these women are likely to present with persistently low UIC despite increased renal iodine clearance [49], making UIC a feasible test in pregnant women with moderate to severe iodine deficiency.

Iodine deficiency predisposes to preeclampsia through defective placental angiogenesis in the first trimester, which leads to ischaemia, atherogenesis, oxidative stress, diminished PlGF production, increased trophoblastic apoptosis and elevated sFlt-1 secretion leading to maternal systemic endothelial dysfunction [50,51]. In addition, as one of the most important exogenous anti-oxidants, the low serum level of iodine leads to oxidative imbalance and further endothelial activation, dysfunction, and more severe manifestation of preeclampsia [52,53].

In the current study, FPG had the third-best AUC for discriminating pre-eclampsia. Interestingly, the optimal cut-off (95 mg/dL) is close to the fasting blood sugar level of 5.3 mmol/L recommended for diagnosis of gestational diabetes by the American Diabetic Association [54]. Therefore, routine screening for diabetes using FPG among pregnant women can also identify women at risk of preeclampsia. This is not surprising, as most markers associated with insulin resistance in the current study, such as dyslipidaemia and high BMI, were significantly higher among cases than controls.

Consistent with other studies [55,56,57], our data seem to suggest that SCH is associated with preeclampsia, as exemplified by T3 and T4 levels within the normal range for both cases and controls, but an elevated mean TSH level for cases of 5.90 ± 2.56 mIU/L, which is well above the recommended pregnancy upper limit of 2.5–3 mIU/L. The underlying cause of SCH in the study population seems to be iodine deficiency, whose sensitivity and specificity are better than those of TSH. This and the high cost of TSH analysis would preclude its routine use as a screening test for preeclampsia in low-resource settings.

The fairly good capacity of a lower percentage of lymphocytes as a predictor of preeclampsia observed in the current study could be attributed to the relative increase in neutrophils. Canzoneri et al. found a significantly higher total leukocyte count among women with severe preeclampsia due to the marked increase in neutrophil numbers: 8.05 ± 4.01 (severe preeclampsia) versus 6.69 ± 2.23 (mild preeclampsia) versus 5.90 ± 1.79 (controls) (*p* < 0.0001) [58]. Preeclampsia is associated with the activation of neutrophils and other leukocytes with enhanced superoxide production and the release of endothelial mediators, such as tumour necrotic factor alpha and interleukin-8, that lead to endothelial dysfunction [59,60].

Consistent with other studies, the risk of preeclampsia was much higher among women with an elevation of both oxidized LDL and triglycerides [61,62]. The oxidized LDL/albumin ratio had a fairly high sensitivity but low specificity, probably because low exogenous antioxidant deficiency may play a significant role in the early stages of preeclampsia, while renal and hepatic injury associated with a significant reduction in albumin levels occur in late and severe preeclampsia. As for serum TSH, the low sensitivity and specificity of the lymphocyte percentage and the oxidized LDL/albumin ratio preclude their potential use as screening tests for preeclampsia.

In the current study, serum selenium levels were markedly lower among cases than controls. Selenium protects thyroid parenchyma against destruction by excessive oxidant, endogenous peroxide, and superoxide molecules produced during thyroid synthesis [63,64]. Furthermore, selenium is a constituent of deiodinase enzymes necessary for converting thyroxine into more potent triiodothyronine [63,64]. This may further explain the higher serum TSH level observed among women with preeclampsia, in addition to their lower iodine nutrition status. Furthermore, selenium is a constituent of glutathione, one of the major antioxidants in circulation, potentially reducing the risk of preeclampsia among women with high selenium intake as most of the normotensive controls [14]. This calls for ensuring adequate iodine and selenium nutrition among the general population, especially pregnant women.

Given the findings in the current study, it is prudent to promote regular consumption of diets rich in selenium, potassium, magnesium, and iodized salt for the general population, but more specifically during pregnancy. In low-resource settings, where screening for incident preeclampsia with efficient but costly tests is less feasible, food frequency questions should be routinely administered during antenatal care to identify women with deficient diets who may require further micronutrient supplementation in addition to iron and folic acid.

### Study Limitations

The current study is limited by the unmatched case-control study design, which precluded the ascertainment of a temporal relationship between the observed values of the biomarkers and preeclampsia and is more prone to bias than a cohort study. The performance of screening tests would be better evaluated with prospective cohort studies, especially for detecting early-onset preeclampsia associated with more perinatal complications [65]. Secondly, the performance of some screening tests may vary according to regional, socio-economic, and ethnic differences, which may affect the universal application of the cut-off values [66]. Furthermore, our study included many variables; hence, requiring a larger sample size for better analysis of how the interactions between several variables can affect the strength of association with preeclampsia.

## 5. Conclusions

The serum potassium/magnesium ratio, obtained from routine laboratory tests, showed a high potential as a biomarker for screening and detecting women at risk of preeclampsia. The urinary iodine concentration, serum TSH, the oxidized LDL/albumin ratio and FPG, and lymphocytosis may be useful in the prediction of women at increased risk of preeclampsia among populations with iodine deficiency, micronutrient malnutrition, and recurrent infections, respectively. Furthermore, the association of the various micronutrients with preeclampsia needs to be explored in a longitudinal study to assess if this association starts early in pregnancy and, if so, the cut-off values that could be used to predict incident preeclampsia.

## Figures and Tables

**Table 1:** Median (25th and 75th percentiles) levels of various analytes of cases and controls.

	Cases	Controls	
Biomarker	Median (25p, 75p)	Median (25p, 75p)	*p*
HDL–C mg/dL	16.0 (12.0, 29.6)	21.5 (12.0, 45.8)	0.080
LDL–C mg/dL	125.0 (87.0, 154.0)	121.0 (67.0, 134.0)	0.003
Oxidized LDL IU/L	167.0 (89.0, 221.0)	82.0 (19.7, 212)	<0.0001
Triglycerides mg/dL	144.5 (84.0, 189.0)	84.0 (67.8, 139.5)	<0.0001
Total Cholesterol mg/dL	145.0 (125.0, 199.0)	126.0 (95.3, 145.2)	<0.0001
Waist Circumference cm	79.0 (72.0, 90.0)	75.0 (70.0, 79.0)	<0.0001
Hip circumference cm	98.0 (87.0, 104.0)	92.0 (85, 97.3)	0.001
BMI kg/M^2^	24.6 (20.8, 28.0)	21.8 (19.0, 25.8)	<0.0001
FPG mg/dL	116.0 (99.0, 180.0)	103.0 (89.0, 125.7)	<0.0001
Cortisol nmol/L	32.9 (18.0, 54.0)	18.0 (18.0, 32.0)	<0.0001
Vitamin C mg/dL	0.45 (0.21, 2.0)	0.60 (0.22, 5.0)	0.002
Selenium µg/L	9.0 (9.0, 17.3)	44.0 (21.0, 102.7)	<0.0001
UIC µg/L	90.0 (78.0, 157.2)	351.0 (299.0, 555.0)	<0.0001
TSH mIU/L	6.3 (4.1, 8.0)	2.5 (0.13, 4.4)	<0.0001
T3 ng/mL	1.32 (1.16, 1.68)	1.16 (1.0, 1.36)	<0.0001
T4 µg/dL	10.9 (9.3, 12.4)	9.8 (8.4, 11.5)	<0.0001
NO µmo/L	2.0 (1.0, 6.0)	20.9 (4.0, 43.3)	<0.0001
OxLDL/Albumin ratio	13.0 (9.0, 16.0)	3.6 (2.0, 12)	<0.0001
Serum Ferritin ng/mL	213.0 (180.0, 345.0)	199.0 (167.0, 340.0)	0.114
GGT U/L	99.0 (88.0, 113.0)	33.0 (11.0, 99.0)	<0.0001
CRP mg/dL	58.5 (39.0, 66.0)	57.0 (12.0, 88.0)	0.024
Lymphocyte %	22.0 (16.0, 25.6)	26.5 (23.5, 38.5)	<0.0001
Serum K^+^ mmol/L	3.6 (2.8, 6.0)	4.0 (3.8, 4.0)	0.149
Serum Mg^2+^ mmol/L	0.12 (0.09, 0.19)	0.97 (0.76, 1.0)	<0.0001
K^+^/Mg^2+^ ratio	28.5 (17.3, 44.3)	4.1 (3.7, 5.3)	<0.0001

OxLDL/Albumin ratio: serum oxidized LDL cholesterol/albumin ratio; UIC: urine iodine concentration; FPG: fasting plasma glucose; K^+^/Mg^2+^: serum potassium/magnesium ratio; TSH: thyroid stimulating hormone.

**Table 2:** Analytes that independently predicted the occurrence of preeclampsia in the study population.

Variable	B	S.E.	Wald	Sig.	Exp(B)	95% C.I. Exp(B)
OxLDL/albumin ratio	0.160	0.061	6.99	0.008	1.174	1.042–1.32
Lymphocytes	−0.282	0.065	19.05	0.000	0.755	0.665–0.856
UIC	−0.013	0.003	16.96	0.000	0.987	0.981–0.993
K^+^/Mg^2+^ ratio	0.160	0.027	35.83	0.000	1.173	1.113–1.236
TSH	0.336	0.132	6.51	0.011	1.400	1.081–1.812
FPG	0.441	0.003	5.12	0.024	0.993	0.986–0.999
Constant	5.61	2.46	5.22	0.022	272.892	

OxLDL/Albumin ratio: serum oxidized LDL cholesterol/albumin ratio; UIC: urine iodine concentration; FPG: fasting plasma glucose; K^+^/Mg^2+^: serum potassium/magnesium ratio; TSH: thyroid stimulating hormone.

**Table 3:** The cut-offs, sensitivity, specificity, and areas under the receiver operating curves of various diagnostic biomarkers for the prediction of preeclampsia.

Analyte	Cut-Off Limit	Sensitivity	Specificity	AUC	95% CI	*p*
K^+^/Mg^2+^	>22	93.0%	95.0%	0.973	0.953–0.993	<0.0001
UIC	<239 µg/L	98.0%	80.0%	0.920	0.893–0.946	<0.0001
FPG	>95 mg/dL	81.2%	91.3%	0.860	0.822–0.897	<0.0001
TSH	>3.9 mIU/L	78.0%	73.0%	0.812	0.771–0.854	<0.0001
Lymphocyte %	<23.5	72.7%	63.2%	0.773	0.729–0.818	<0.0001
OxLDL/Alb Ratio	>7.0	80.0%	65.0%	0.746	0.695–0.797	<0.0001
Selenium	<20 µg/L	79.3%	96.0%	0.885	0.843–0.926	<0.0001
Nitric oxide	<10 µg/L	60%	94%	0.784	0.730–0.837	<0.0001

OxLDL/Alb Ratio: serum oxidised LDL cholesterol/albumin ratio; UIC: urine iodine concentration; FPG: fasting plasma glucose; K^+^/Mg^2+^: serum potassium/magnesium ratio; TSH: thyroid stimulating hormone.

## Data Availability

The raw data supporting the conclusions of this article will be made available by the authors on request.

## Abbreviations

The following abbreviations are used in this manuscript:

Alb	albumin
BMI	body mass index
Ca^2+^	calcium ions
CRP	C-reactive protein
GGT	gamma glutamate transferase
HDL	high density lipoprotein
HDL-c	high density lipoprotein cholesterol
LDL	low density lipoprotein
LDL-c	low density lipoprotein cholesterol
L/WBC%	lymphocyte percentage
Mg^2+^	magnesium ions
NO	nitric oxide
OxLDL	oxidized low density lipoprotein
K^+^	potassium ions
TSH	thyroid stimulating hormone
T3	triiodothyronine
UIC	urinary iodine concentration
